# Micronutrients in COVID-19 Positive Pregnancies

**DOI:** 10.7759/cureus.10609

**Published:** 2020-09-23

**Authors:** Pinar Yalcin Bahat, Merve Aldikactioglu Talmac, Ayşegul Bestel, Nura F Topbas Selcuki, Zelal Aydın, İbrahim Polat

**Affiliations:** 1 Obstetrics and Gynaecology, Kanuni Sultan Süleyman Training and Research Hospital, University of Health Sciences, Istanbul, TUR; 2 Obstetrics and Gynaecology, Health Sciences University, Istanbul Sisli Hamidiye Etfal Training and Research Hospital, Istanbul, TUR

**Keywords:** 25-oh vitamin d, serum vitamin b12, zinc, covid-19 in pregnancy, micronutrients

## Abstract

Pregnant women are considered among the high-risk population for COVID-19. Therefore, research for methods of treatment and prevention of COVID-19 positive pregnancies carries an importance. The aim of this study was to measure serum 25(OH)D, vitamin B12, and zinc levels in COVID-19 positive pregnant women to evaluate the role of these micronutrients in treatment and prevention. A total of 44 COVID-19 positive pregnant women who were hospitalized and treated at a tertiary clinic were included in this study. The mean serum 25(OH)D level was measured to be 9.70 ± 59.14. The mean serum zinc level was 62.58 ± 2.63, and the mean serum vitamin B12 level was 295.55 ± 302.48. All these variables were significantly lower than the accepted cut-off values (p < 0.001). These low values might have contributed to a deficiency in their immune response and thus made these patients susceptible to COVID-19 infection. Supplementation of micronutrients during the pandemic could be beneficial during pregnancy for prevention.

## Introduction

Coronavirus family has long been a well-known source of infection causing diseases like common cold, severe acute respiratory syndrome (SARS), and Middle East respiratory syndrome (MERS). Novel coronavirus 2019 (SARS-CoV-2) is a newly discovered virus from the coronavirus family, which is the infection source of the COVID-19 pandemic [1]. COVID-19 enters the host cells and triggers an immune response, which includes the production of proinflammatory cytokines, activation of T cells, CD4, and CD8+ T cells [2]. The severe forms of the disease leading to acute respiratory distress syndrome have been attributed to excessive production of proinflammatory cytokines also called ‘cytokine storm’ [3]. Since the outbreak, extensive research has been put into understanding the action mechanism of COVID-19 in hope of finding a cure and a vaccine. However, there are still unknowns especially about the potential protective factors against the virus.

Vitamins and minerals, also known as micronutrients, are key factors in maintaining a healthy immune system and therefore are widely used as supplements for protection against bacterial and viral infections [4]. Zinc, 25 hydroxyvitamin D (25(OH)D), and vitamin B12 belong to this family of micronutrients.

Zinc is an essential micronutrient that is involved in cell proliferation, differentiation, RNA and DNA synthesis [5], as well as cell structures and cell membrane stabilization [6]. There is also strong evidence between zinc deficiency and several infectious diseases, such as malaria, HIV, tuberculosis, measles, and pneumonia. Zinc is also involved in the modulation of the proinflammatory response by regulation of inflammatory cytokines and in controlling oxidative stress [7]. Similarly, 25(OH)D has anti-infective, anti-inflammatory, and immunomodulant functions. It contributes to the maintenance of the cell’s physical barrier integrity, enhanced activity of innate immunity through macrophages and monocytes, and T cells [8]. Lastly, vitamin B12 is a water-soluble vitamin. It is a cofactor in DNA synthesis and is known to inhibit viral replication in the host cells [9].

The aim of this study was to measure serum 25(OH)D, vitamin B12, and zinc levels in COVID-19 positive pregnant women to evaluate the role of these micronutrients in the prevention and to evaluate the possible cause between the blood levels of micronutrients and the COVID-19 infection.

## Materials and methods

This case-control study was conducted in a tertiary referral hospital between April and June 2020. The study protocol was approved by the institution’s Ethics Committee (2020.05.25) and registered to ClinicalTrials.gov (NCT04407572). Written informed consent was obtained from all participants before their enrollment in the study.

Study population

A total of 44 COVID-19 positive pregnant women were included in the study. Pregnant women whose PCR test was positive for COVID-19, pregnancies older than eight weeks of gestation, and women who did not receive any antibacterial or antiviral treatment during the past three months or did not receive any 25(OH)D, vitamin B12, and zinc supplements during their pregnancy were included in the study. Women with known renal disease, rheumatic disease, diabetes mellitus type 1, acquired immune deficiency syndrome, and those using immunosuppressants were excluded. Complicated pregnancies such as ectopic pregnancy, scar pregnancy, or hydatidiform mole were also excluded.

A detailed medical history was obtained. All patients underwent a physical and obstetrical examination with ultrasound and received a thorax CT. In addition to routine blood tests, D-dimer levels and ferritin levels were also determined. Gestational age was estimated based on the last menstrual period. If the date of the last menstruation was unknown, then crown-rump length (CRL) was used for calculation.

Laboratory analysis

On the day of admission, blood samples were taken from a peripheral vein using one dry tube and one tube with ethylenediaminetetraacetic acid (EDTA). Tubes were submitted to the hospital’s central laboratory for determination of 25(OH)D, zinc, and vitamin B12 levels. Quantitative determination of 25(OH)D was performed with competitive chemiluminescent immunoassay (CLIA) using LIAISON® 25 OH Vitamin D TOTAL Assay (DiaSorin S. p. A., Saluggia, Italy). Vitamin D deficiency was defined as serum vitamin D levels of less than 20 ng/mL, insufficiency as 21-29 ng/mL, and sufficiency as 30-100 ng/mL according to recent Clinical Guidelines Committee. Recommended levels are higher than 30 ng/mL for specific groups, such as in pregnant women. The mean of 19.9 ng/mL (SD: 0.948 ng/mL and an intra-control CV of 4.8%) was the referred reproducible value to measure the 25(OH)D. For the same parameter, the intermediate precision was 19.9 ng/mL (SD: 1.23 ng/mL and an intra-control CV of 6.2%) [10]. Access Immunoassay System (© 2020Beckman Coulter Inc., Brea, CA, USA) was used to quantitate serum vitamin B12 levels. A normal range of 100-350 pmol/L serum vitamin B12 was calculated with a 95% confidence interval [11]. For zinc quantification, atomic absorption spectroscopy was used (PerkinElmer® Inc., Waltham, MA, USA). Current cut-off levels for zinc deficiency are <8.7 μmol/L (afternoon, non-fasting) and for non-fasting women of reproductive age (aged 18-49 years; WRA, <10.1 μmol/L (morning) and <9.0 μmol/L (afternoon)) [12]. All calculations followed the best clinical practice.

Statistical analysis

Statistical Package for Social Sciences 23.0 (IBM Corp., Armonk, NY, USA) was used for statistical analysis. Descriptive statistical methods (mean, standard deviation, frequency) and their associated 95% confidence intervals of study variables were presented. One sample t-test was performed to evaluate the difference between accepted cut-off values and the means of our study groups regarding B12, zinc, and 25(OH)D levels. P-value <0.05 was considered for statistical significance.

## Results

A total of 44 COVID-19 positive pregnant women were included in the study. The mean age was 28.57 ± 7.77 years. When pregnancies were divided into trimesters, it was observed that disease rate in our cohort increased with increasing trimester: 8.8% (n = 5) was in the first trimester, 27.2% (n = 12) were in the second trimester, and 61.3% (n = 27) were in the third trimester. The mean gravidity was 2.75 ± 1.74, and the mean parity was 1.32 ± 1.37 (Table 1).

**Table 1:** Patient’s demographic data

Demographic characteristics		Mean and standard deviation, n (%)	95% CI lower	95% CI upper
Age (years)		28.57 ± 7.77	26.45	30.80
Gravida (n)		2.75 ± 1.74	2.27	3.30
Parity (n)		1.32 ± 1.37	0.93	1.73
Zinc (70-114 μmol/L)		62.58 ± 2.63	54.81	70.18
Vitamin D (20-80 ng/mL)		9.70 ± 59.14	8.06	11.54
Vitamin B12 (197-771 pmol/L)		295.55 ± 302.48	2.21	3.93
Trimester	1	5 (11.4%)	2.3	22.7
	2	12 (27.3%)	13.6	40.9
	3	27 (61.4%)	47.7	75.0
CT	0	17 (38.63%)	25.0	54.5
	1	12 (27.27%)	13.6	40.9
	2	12 (27.27%)	15.9	40.9
	3	3 (6.81%)	0.0	13.6
Hydroxychloroquine treatment	Yes	18 (40.90%)	27.3	56.8
	No	26 (59.10%)	43.2	72.2
D-Dimer levels (ng/mL)		1.72 ± 0.91	1.47	2.00
Ferritin levels (ng/mL)		64.10 ± 187.62	3.15	12.42
Duration of hospital stay (days)		6.63 ± 1.70	-	-

When thorax CTs were evaluated, 38.63% (n = 17) did not reveal any pathology, 27.327% (n = 12) showed mild changes, and in 27.27% (n = 12) moderate and in 6.81% (n = 3) severe findings could be observed. Majority of the patients in the study presented with mild to moderate CT findings. Approximately 40.90% were treated with hydroxychloroquine, whereas 59.10% were monitored actively without any medical intervention. At the time of admission, the mean serum D-dimer level was 1.72 ± 0.91 and the ferritin level was 64.10 ± 187.62. The mean duration of stay at the hospital was 6.63 ± 1.70 (3-11) (Table 1). While the minimum stay in the hospital was 3 days and the maximum stay was 11 days.

The mean serum 25(OH)D level was measured to be 9.70 ± 59.14. The mean serum zinc level was 62.58 ± 2.63, and the mean serum vitamin B12 level was 295.55 ± 302.48 (Table 1). In one sample t-test analysis, all these variables were significantly lower than the accepted cut-off values (p-value <0.001): 25(OH)D: 30, zinc: 10.1, and B12: 100.

## Discussion

Understanding the pathogenesis of SARS-CoV-2 has been challenging, and it has not yet been clarified. However, COVID-19 affects the immune system in many different steps in the disease process [13]. Micronutrients are among these immune supplements, and their efficacy has already been shown with other viral infections [14].

Previous studies conducted with pregnant women and women in the postpartum period have shown the efficacy of micronutrients in fight against viral infections. Especially, the inhibitory effects of 25(OH)D, vitamin B12, and zinc, due to their safe use during pregnancy, on viral pathogenesis have already been shown. For example, in studies of H1N1 prior to the finding of a vaccine, the protective effects of high levels of vitamin B12 on the newborns ingested through breast milk were observed [15]. Another study has shown that high levels of maternal serum 25(OH)D levels during pregnancy and lactation were associated with lower rates of acute respiratory infections of the newborns [16]. Additionally, it has been shown that serum zinc levels correlate positively with better immune response in pregnant women against infections [17].

It is known that 25(OH)D plays an essential role in immune answer and its modulation [18,19]. In a study conducted by Ilie et al., in countries that had a severe course of COVID-19 such as Spain, Italy, and the United Kingdom, mean serum 25(OH)D levels are low [20]. Another study showed that patients with high serum vitamin D levels sustained less lung damage. According to a study by Alipio, lower rates of multiple organ failure were observed among patients with higher levels of serum 25(OH)D [21]. From the existing evidence, it can be concluded that the suppression of viral replication by 25(OH)D is effective in the prevention and also responsible for a mild course of COVID-19 [22]. In our study, serum 25(OH)D levels of the COVID-19 positive pregnant women were under the cutoff value. The reason for these low values could be pregnancy, less exposure to sunlight, and insufficient nutrition. The low levels of serum 25(OH)D, in accordance with the existing data, might have caused susceptibility to COVID-19. 

Zinc plays an important role in the pathogenesis of viral infections. The correlation between serum zinc levels and the severity of infections has already been shown [7]. Anosmia, a common symptom of COVID-19, is also seen in zinc deficiency [23]. In three women, included in the study, with very low levels of serum zinc, anosmia was among the symptoms. Shittu and Afolami advocated in their study that adding zinc supplements to the treatment regimen with chloroquine in COVID-19 patients enhanced the antiviral action mechanism of the chloroquine and efficacy of the treatment [24]. According to another study, it has been suggested that zinc could possess protective effects through reducing inflammation, improving mucociliary clearance, preventing ventilator-induced lung injury, and modulating antiviral and antibacterial immunity [25]. Therefore, zinc can be used both as preventative medication and adjuvant therapy of COVID-19. Similar to the results with 25(OH)D, zinc levels were significantly low in our patient cohort, which might have played a negative effect on their immunity making these patients susceptible during the pandemic.

In vivo studies of vitamin B12 have shown that vitamin B12 acts as a natural inhibitor of viral replication of the hepatitis C virus (HCV) [26]. In another study, it was shown that vitamin B12 and folate levels affect the human papillomavirus methylation process and low serum levels increase malignancy [27]. In our study, all serum vitamin B12 levels were significantly low can be making them more prone to COVID-19.

There are several limitations to this study. First, there are difficulties in the measurement of serum 25(OH)D levels in pregnant women, which might have led to the lower levels observed in this study. Additionally, a limited number of patients and a lack of a control group were further limitations. However, due to the need for cumulative data concerning the COVID-19 pandemic, we believe that our results will be of importance for further studies on prevention and adjuvant therapy. 

## Conclusions

The level of serum micronutrients in pregnant women with COVID-19 was lower than the cut-off values. These low values might have contributed to a deficiency in their immune response and thus made these patients susceptible to COVID-19 infection. Supplementation of micronutrients during the pandemic could be beneficial during pregnancy for prevention. However, further studies are needed to show their effects on COVID-19 infection.
